# NumbL is essential for *Xenopus* primary neurogenesis

**DOI:** 10.1186/1471-213X-13-36

**Published:** 2013-10-14

**Authors:** Frank Nieber, Marie Hedderich, Olaf Jahn, Tomas Pieler, Kristine A Henningfeld

**Affiliations:** 1 Institute of Developmental Biochemistry, University of Goettingen, Goettingen, Germany; 2 Center for Nanoscale Microscopy and Molecular Physiology of the Brain (CNMPB), Goettingen 37077, Germany; 3 Max Planck Institute of Experimental Medicine, Proteomics Group, Hermann-Rein-Str. 3, Goettingen 37075, Germany

**Keywords:** Numb, Notch, Primary neurogenesis, Neurogenin, Neuronal differentiation, Xenopus

## Abstract

**Background:**

Members of the vertebrate Numb family of cell fate determinants serve multiple functions throughout early embryogenesis, including an essential role in the development of the nervous system. The Numb proteins interact with various partner proteins and correspondingly participate in multiple cellular activities, including inhibition of the Notch pathway.

**Results:**

Here, we describe the expression characteristics of *Numb* and *Numblike* (*NumbL*) during *Xenopus* development and characterize the function of *NumbL* during primary neurogenesis. *NumbL*, in contrast to *Numb*, is expressed in the territories of primary neurogenesis and is positively regulated by the Neurogenin family of proneural transcription factors. Knockdown of NumbL afforded a complete loss of primary neurons and did not lead to an increase in Notch signaling in the open neural plate. Furthermore, we provide evidence that interaction of NumbL with the AP-2 complex is required for NumbL function during primary neurogenesis.

**Conclusion:**

We demonstrate an essential role of NumbL during *Xenopus* primary neurogenesis and provide evidence for a Notch-independent function of NumbL in this context.

## Background

Numb type proteins define an evolutionary conserved class of adaptor proteins that have been implicated in a variety of cellular activities, including the regulation of cell polarity, cell migration, as well as target protein endocytosis and ubiquitination [1-4]. Numb was first identified as a membrane-associated protein that is asymmetrically segregated to only one daughter of the *Drosophila* sensory organ precursor cells (SOP) in the peripheral nervous system [5,6]. In this context, Numb functions as an intrinsic cell fate determinant and the daughter cell that inherits Numb adopts a different cell fate from the other daughter cell [6]. The ability of Numb to direct specific binary cell fate decisions in the SOP lineage was subsequently attributed to its ability to antagonize the Notch signaling pathway [6-8].

**Figure 1:**
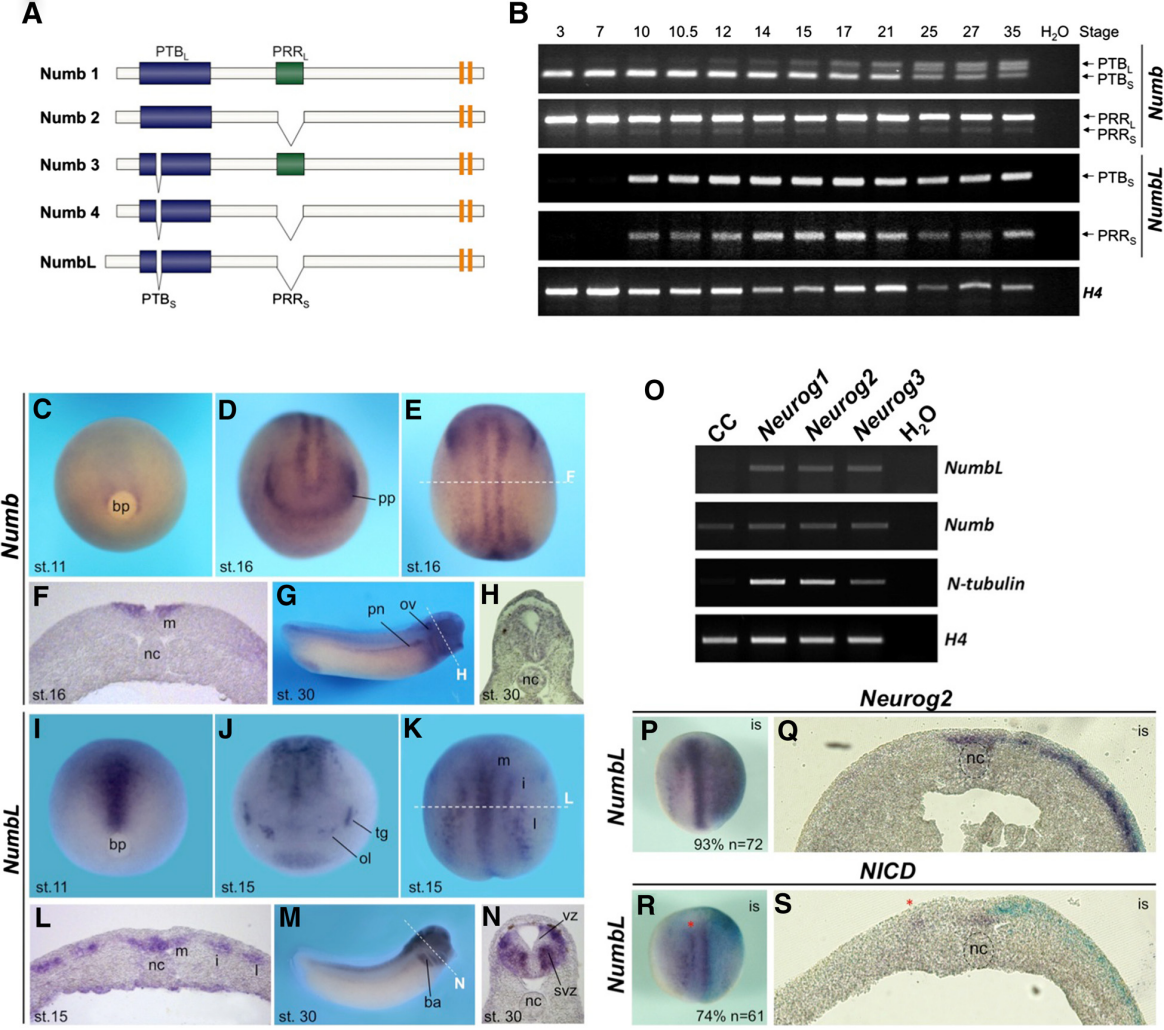
**Expression and regulation of *Xenopus Numb *and *NumbL*. (A)** Schematic representation of the mouse Numb proteins. The EH motifs are in orange. **(B)** RT-PCR analysis using *X. tropicalis* cDNA. Primer pairs used spanning the putative insert regions of the isoforms are indicated on the right side. Arrows indicate the expected PCR products. **(C**-**N)** Whole mount in situ expression analysis of *X. laevis* embryos using a *X. tropicalis Numb* or *X. laevis NumbL* antisense RNA probe. Stage 11 embryos shown in C and I are a blastopore view with the dorsal side up. Stage 15/16 embryos are shown in an anterior view **(D** and **J)** a dorsal view anterior up **(E** and **K)**, as well as in transversal sections **(F** and **L)**. Stage 30 embryos are shown in a lateral view **(G** and **M)** and as a transversal section at the level of the hindbrain **(H** and **N)**. The plane of the transversal sections of E, G, K and M are indicated with a white dotted line. **(O)** RT-PCR analysis of *X. laevis* animal caps injected with 20 pg *Neurog1-3* mRNA. Explants were cultured until control siblings reached stage 15. H_2_O represents a negative control where no RNA was added to the RT reaction. **(P**-**S)** Whole-mount in situ hybridization of stage 14 embryos injected with *Neurog2* (20 pg) or *NICD* (50 pg) mRNA together with *β-Gal* (75 pg) mRNA (light blue staining). The injected side is on the right and embryos are shown as a dorsal view. The red asterisk in R and S marks the intermediate stripe that is inhibited on the NICD-injected side. bp, blastoporous; nc, notochord; m, medial stripe; i, intermediate stripe; l, lateral stripe; ba, branchial arches; ol, olfactory placode; ov, otic vesicle; pn, pronephros; pp, panplacodal primordium; vz, ventricular zone; svz, subventricular zone.

In vertebrates, there are two closely related genes that are homologues of *Drosophila Numb*, namely *Numb* and *Numblike* (*NumbL*) [9-11]. Furthermore, there are four predominant vertebrate isoforms of Numb, which are generated by alternative splicing (Figure 1A). Splice variants for NumbL have not been described. The Numb proteins are characterized by an amino-terminal phosphotyrosine binding (PTB) domain, a centrally located proline rich repeat (PRR) domain and two EPS15 homology (EH) motifs in the C-terminus [12]. The Numb isoforms differ from each other by the presence or absence of an 11 amino acid insert in the (PTB_L_ or PTB_S_), or a 48 amino acid insert in the PRR domain (PRR_L_ or PTB_S_) [13]. Two additional truncated isoforms of Numb were identified in tumor cells but are expressed at low levels in normal tissue [14]. The Numb protein PTB domain serves as an interaction domain for multiple proteins and controls subcellular localization [12]. Numb PTB_S_ isoforms localize to the plasma membrane, while Numb PTB_L_ isoforms are cytoplasmically localized [12]. The PRR domain contributes to proliferative and differentiation activities of Numb. Whereas Numb isoforms containing the PRR_S_ domain promote differentiation, Numb PRR_L_ isoforms promote proliferation of a variety of cell types [12]. While *Numb* and *NumbL* are structurally related, they differ in their expression. *Numb* is broadly expressed throughout the developing mouse and chick embryos, *NumbL* is highly enriched in the developing central nervous system [10,11,15].

There are several lines of evidence supporting an evolutionary conservation of Numb function. Vertebrate Numb proteins are asymmetrically distributed in dividing neural progenitors [10,15-18] and can rescue the Numb mutant phenotype in *Drosophila*[9,10]. Moreover, mammalian Numbs have been demonstrated to inhibit Notch signaling in the developing *Drosophila* outer optic anlage [19] and chick neuroepithelium [15]. Mechanistically, Numb can inhibit Notch signaling by promoting endocytosis of the Notch receptor, or ubiquitination and subsequent degradation of the active form of the Notch receptor, NICD (Notch intracellular domain) [3,4,20,21].

*Numb* and *NumbL* were found to act in a partially redundant manner and to be essential for neural development in the mouse [22-25]. However, the function of the Numbs during the development of the nervous system remains controversial as, depending on the time of gene inactivation, the phenotypes observed suggest a role for Numb and NumbL in both progenitor maintenance and differentiation. Moreover, the depletion of progenitor cells observed upon inactivation of Numb and NumbL in mice prior to or after the onset of neurogenesis [23,25] is inconsistent with Numb proteins acting as Notch inhibitors, as Notch signaling is also required for progenitor cell maintenance [26,27]. In addition to their ability to negatively regulate the Notch pathway, the Numb proteins can influence other pathways such as the one regulated by Hedgehog or p53, which may also contribute to the observed phenotypes [21,28].

As the molecular mechanism of vertebrate Numb protein function during early development of the nervous system remains unclear, we wished to study their activities in the context of *Xenopus* primary neurogenesis. In *Xenopus*, the first neurons, termed primary neurons, are born in the posterior neural plate shortly after gastrulation in three bilateral stripes [29]. The expression of the Neurogenin (Neurog) family of proneural bHLH factors prefigures these domains, and, through the activation of downstream differentiation factors, promotes neuronal differentiation in a subset of cells in the neuroepithelium [30,31]. The proneural proteins also activate lateral inhibition mediated by the Notch signaling pathway, which restricts the number of cells that undergo neuronal differentiation [32]. Correspondingly, inhibition of the Notch pathway, either by chemicals or dominant-negative constructs, increases the density of neuron formation within the territories of primary neurogenesis [33,34].

Here, we describe a comparative expression analysis of *Numb* and *NumbL* during early development of *Xenopus*. *Numb* is expressed weakly and ubiquitously in early cleavage and gastrula stage embryos. At neurula stages, *Numb* transcripts are enriched in a bilateral longitudinal domain and in the anterior panplacodal region. In contrast, *NumbL* is strongly expressed in the midline starting at late gastrula stages; at the open neural plate stage, expression is additionally present in the territories of primary neurogenesis. *NumbL,* but not *Numb,* is positively regulated by the Neurogenin-type proneural factors and inhibited by the Notch pathway. Knock-down experiments demonstrate that NumbL function is essential for neuronal differentiation. We also provide evidence that the inhibition of neuronal differentiation is not due to a deregulation of the Notch signaling pathway. Finally, we show that NumbL interacts with the endocytotic machinery in *X. laevis* embryos and provide evidence that suggests that this interaction is essential for its function.

## Methods

### Constructs

*X. laevis NumbL* was obtained by screening a tadpole head λZAP library with a partial cDNA sequence (RZPD, image 6643616) using the ECL Direct Nucleic Acid Labeling and Detection System (Amersham Bioscience). The isolated *NumbL* clone (KF589315) in the pBKCMV vector (Stratagene) contains 3158 bp including the complete coding sequence. For overexpression, *X. laevis NumbL* was PCR amplified using the following primers (for 5′-CGGGAATTCTGCCCATTCAGT-3′ and rev 5′-CGGCTCGAGCTAGTGCTGGCA-3′) and cloned EcoRI/XhoI into MTpCS2+ [35]. A clone containing the entire opening reading frame of *X. tropicalis Numb3* in pCS107 was purchased from MRC gene services (Tegg132l24/CR942503). Comparison of the *X. laevis* and *X. tropicalis* NumbL and Numb amino sequences with the corresponding mouse sequences are depicted in Additional file 1: Figure S1 and Additional file 2: Figure S2. *X. laevis NumbL* mutants were prepared by site directed mutagenesis of the *MT-NumbL*pCS2+ clone using the Quick Exchange XL Kit (Stratagene) and the following primers with introduced mutations indicated in lower case letters: S291A for 5′- GCTGGTGAGACAGGGAgCCTTTCGTGGATTCC-3′ and rev 5′- GGAATCCACGAAAGGcTCCCTGTCTCACCAGC-3′, S310A for 5′-CCTTTTAAACGGCAGCTTgCGCTGAAACTCAATGAGC-3′ and rev 5′- GCTCATTGAGTTTCAGCGcAAGCTGCCGTTTAAAAGG-3′, NL DLA for 5′-AGGAAGTGGACCtGgcTGAAGCTCAATGGG-3′ and rev 5′- CCCATTGAGCTTCAgcCaGGTCCACTTCCT-3′). *Mouse NumbL*pBS [11] and *Numb1-4*pEN [13] were subcloned for overexpression EcoRI/XhoI in pCS2+. The following overexpression constructs have been described previously: *Ngnr-1* (*Neurog2*) [30]; *X. tropicalis Neurog 1–3*[31]; *Delta*^*STU*^ and *LacZ*[32]; *Su(H)*^*DBM*^[33]; *NICD*[36]; Noggin [37]. Unless otherwise indicated, the constructs contained *X. laevis* cDNA sequences.

### Embryo culture and microinjection

Embryos were obtained from *X. laevis* and *X. tropicalis* by HCG induced egg-laying using standard techniques and staged according to Nieuwkoop and Faber [38]. Capped mRNA for microinjection was in vitro transcribed from NotI linearized constructs (SP6 mMessage mMachine™ Ambion) and purified using the RNeasy kit (Qiagen). Embryos were injected in one or both blastomeres of the two-cell stage with a volume of 4 nl. Antisense morpholinos were obtained from Gene Tools LLC. *NumbL MO*: 5′-GCGCAGTAGTTGATGTTTGCCCTCA-3′; *NumbL* mmMO: 5′-GCCCAGTACTTCATCTTTGGCGTCA-3′.

### Whole mount in situ hybridization and histology

X-Gal staining and whole mount in situ hybridizations were performed as described [39], using antisense RNA probes labeled with digoxigenin-11-UTP. For the double in situ hybridization *EpiK* was labelled with fluorescein and stained with FastRed (Roche) and *X. tropicalis Numb* with digoxygenin and NBT/BCIP. The pH3 assay was performed as described [40]. Embryos were embedded in gelatin/albumin and sectioned on a Leica Vibratome VT1000M (30 μm). The following marker constructs were used for preparation of antisense RNA probes, enzymes used for linearization and polymerases are indicated: *X. laevis NumbL*pBKCMV (EcoRI, T7); *Sox3* pBSK (EcoRI, T7); *ESR3/7* RZPD/imagenes pCMV-Sport6 (BC072958) (Sal1, T7); *EpiK*pGEM-T (*XK81*)(EcoRI/SP6); *ESR8*pBS-CMV (EcoR1, T7)*; ESR10*pBSK (SalI, T7); *Neurog2* (*Ngnr-1*) [30]; *ESR1*[33]; *N-tubulin*[41]; *MyT1*[42]; *NCAM*[43]; *Pak3*[44]; *NeuroD*[45] and *X. tropicalis Numb3*pCS10*7* (CR942503). Unless otherwise indicated, the constructs contained *X. laevis* cDNA sequences.

### Animal cap dissection and RT-PCR

Embryos were bilaterally injected at the two-cell stage, animal caps were dissected from stage 8 – 9 embryos and cultured until sibling control embryos reached the desired stage. Total RNA was extracted from animal caps or whole embryos using phenol/chloroform (PeqGold, Peqlab). cDNA was prepared using random hexamer primers. Primers used for PCR: *Xt Numb-PTB* (for 5′-AGGAATCAAGAGGGATGCAC-3′ and rev 5′-CATCCACAACTCGAAGTCCA-3′), *Xt Numb-PRR* (for 5′-ACACTTTCAGCATGCCACCT-3′ and rev 5′-CTTCTTCAAGCCAACGGTCT-3′), *Xt NumbL-PTB* (for 5′-AGGAGTCGAGGGGAATGC-3′ and rev 5′-ACAACACGCAGCCCATCA-3′), *Xt NumbL-PRR* (for 5′- CTTCCACCACAACCAATGC-3′ and rev 5-TCCTCAAGCCAGCGTTCC-3′), *Xt H4* (for 5′-CGGGATAACATTCAGGGTATCACT-3′ and rev 5′-ATCCATGGCGGTAACTGTCTTCCT-3′). *Xl NumbL* (for 5′-AAGCCATCCTCTGGGTTTCT-3′ and rev 5′-CTGTCACCCCACACTCCTTT-3′), *Xl Numb* (for 5′-GAGGATATGGCAAGGCAAAA-3′ and rev 5′-AACCACAGCCAGTCCAGTTC-3′), *Xl N-tubulin* (for 5′-ACACGGCATTGATCCTACAG-3′ and rev 5′-AGCTCCTTCGGTGTAATGAC-3′), *Xl H4* (for 5′-CGGGATAACATTCAGGGTATCACT-3′ and rev 5′-ATCCATGGCGGTAACTGTCTTCCT-3′).

### Luciferase assay

For Luciferase assays, the Dual Luciferase Reporter Kit (Promega) and a Centro LB960 luminometer (Berthold Technologies) were used. As Notch reporter construct, 5 pg of the Hes1 promotor (−194 to +160) fused to Firefly luciferase were co-injected [46] with 5 pg of CMV-Renilla luciferase construct for normalization. *NICD* and *Noggin* mRNA (each 50 pg) were injected in the presence or absence of *NumbL* mRNA (100 pg). Embryos were cultivated until open neural plate stage and then frozen in liquid nitrogen in batches containing at least ten embryos. Normalized luciferase activity was measured according to the manufacturer’s protocol.

### Tandem affinity pull-down and mass spectrometry analysis

For tandem affinity purification of NumbL, 2000 *X. laevis* embryos were injected in both blastomeres at the 2-cell stage with *NumbL-CTap* or *CTap* mRNA (500 pg/injection), grown until stage 15 and then shock frozen in liquid nitrogen. The tandem affinity purification was essentially performed as described [47]. All steps were performed at 4°C, 10 μl lysis buffer per embryo were used. After the 2nd pulldown, proteins were eluted in 100 μl 2× SDS loading buffer and 2 × 25 μl were loaded on a pre-cast NuPAGE Bis-Tris 4-12% gradient gel (MES buffer system; Invitrogen) for separation of the candidate proteins. Protein bands were visualized by Colloidal Coomassie or silver staining. Candidate bands were excised and subjected to tryptic in-gel digest followed by mass spectrometric protein identification. To obtain complementary identification data, extracted peptides were prepared for analysis on an Ultraflex MALDI-TOF/TOF mass spectrometer (Bruker) and additionally analyzed by LC-MS/MS on a LCQ Deca XP Plus ESI ion trap mass spectrometer (Thermo) as described [48,49]. By using the Mascot Software 2.3 (Matrix Science; see Patzig et al. [49] for parameter settings), data files were searched against the NCBInr primary sequence database (version 20130509) restricted to the taxonomy *Xenopus laevis* (17,413 sequences) and also without taxonomy restriction (25,501,115 sequences) to cover all protein sequences of the *Xenopus* genus. The minimal requirement for accepting a protein as identified was at least one peptide sequence match above identity threshold in coincidence with at least 20% sequence coverage in the peptide mass fingerprint (MALDI data) or at least two peptide sequence matches above identity threshold (ESI data).

## Results

### *NumbL* is expressed in the areas of primary neurogenesis

Mammalian *Numb* and *NumbL* have been shown to play essential roles during early development, particularly in the context of the nervous system [12]. However, their exact functional roles and molecular mechanisms of action have been under debate. In a first approach to gain insight into the function of the Numbs during early *Xenopus* development, a comparative expression analysis was undertaken. Four predominate isoforms of mammalian Numb, which are generated by alternative splicing, have thus far been described [13,50]. Therefore, to analyze the expression of the *Numbs*, RT-PCR analysis on staged embryos was performed as it can distinguish between the different isoforms of Numb (Figure 1A and 1B). *X. tropicalis* cDNA was used, as genomic sequence information was available allowing the design of primer sets spanning the putative insert regions of the splice variants.

Starting at gastrula stages and throughout all later *X. tropicalis* developmental stages analyzed, primer pairs spanning the PTB and PRR insert afforded a single amplicon for *NumbL* (Figure 1B). Based on the size and subsequent sequence verification of the isolated PCR product, the *NumbL* amplicons encode a transcript for a protein lacking both inserts (PTB_S_/PRR_S_)*.* In contrast, multiple amplicons were observed for Numb, which correspond to the described PTB_S_/PTB_L_ and PRR_S_/PRR_L_ isoforms. Interestingly, with regard to the PTB domain, a switch in *Numb* isoform expression occurs during embryogenesis. In early cleavage stages, maternal transcripts encode for *Numb* PTB_S_ isoforms. However, starting at the end of gastrulation (stage 12), increasing levels of *Numb PTB*_*L*_ transcripts are detected concomitant with a decrease in *Numb PTB*_*S*_ transcripts. The additional middle amplicon observed with primers spanning the Numb PTB domain could not be sequence verified but its presence may indicate an additional, as of yet, unknown Numb isoform. Throughout the developmental stages analyzed, only low but stable levels of PRR_S_ transcript were detected, indicating that the majority *Numb* isoforms contains the PRR insert. Taken together, from early cleavage stages through neurula stages of *X. tropicalis* development, *Numb3* (PTB_S_ and PRR_L_) is the most predominant *Numb* transcript expressed and at later stages *Numb1* (PTB_L_ and PRR_L_) becomes increasingly abundant.

*Numb* and *NumbL* are expressed in partially overlapping expression patterns in the mouse [10,11]. Thus it is anticipated that they have common as well as distinct functions that correlate with their specific expression patterns. To evaluate their potential roles in the development of the nervous system in *X. laevis* embryos, a detailed comparative spatial expression analysis of *Numb* and *NumbL* by whole mount in situ hybridization was performed (Figure 1C-N). As a *X. laevis* clone for *Numb* was not available, an antisense *X. tropicalis* RNA probe was used for cross-species in situ hybridization in *X. laevis* embryos. *Numb* is expressed at very low levels throughout the *X. laevis* embryo during early cleavage (not shown) and late gastrula stages (Figure 1C). The weak ubiquitous staining observed at these and later stages was confirmed by comparing the expression of a sense RNA probe (Additional file 3: Figure S3). At open neural plate stages, *Numb* is weakly detected throughout the ectoderm and strongly expressed in a horse-shoe shaped domain in the anterior neural plate that marks the panplacodal primordium [51] as well as in a single longitudinal domain on both sides of the midline (Figure 1D and E). Double whole mount in situ hybridization with *Numb* and *Epidermal Keratin* (*EpiK*), which marks the nonneural ectoderm, indicates that *Numb* expression is directly adjacent to *EpiK* and thus marks the neural plate border (Additional file 4: Figure S4). At stage 30, *Numb* transcripts are weakly expressed throughout the embryo but enriched in the anterior region of the embryo, including the brain, and throughout the spinal cord. Transcripts of *Numb* are also found in the otic vesicle and the pronephros (Figure 1G).

In contrast to *Numb*, *NumbL* is first strongly and specifically expressed in a domain starting above the dorsal blastopore lip at mid-gastula (stage 11), which extends anteriorly during late gastrula stages (Figure 1I). At the open neural plate stage, *NumbL* is detected along the midline and in the territories of primary neurogenesis, including the three longitudinal domains in the posterior neural plate. *NumbL* is also present in the neurogenic trigeminal and olfactory placodes (Figure 1J and K). In addition, there is an oval expression domain of *NumbL* in the ventral anterior region of the embryo (Figure 1J). At tailbud stages, *NumbL* continues to be expressed in the nervous system and is detected throughout the brain, spinal cord, eye and branchial arches (Figure 1M). To confirm that difference observed in the expression of *Numb* and *NumbL* in *X. laevis* embryos was not due to using a cross-species *X. tropicalis Numb* antisense probe, the *X. tropicalis Numb* and *X. laevis NumbL* antisense probes were also used for a whole mount in situ hybridization in *X. tropicalis* embryos (Additional file 5: Figure S5). The expression patterns obtained with both antisense probes correlated to those observed in *X. laevis* embryos.

The tissue-specific differences in *NumbL* and *Numb* expression in the neuroectoderm of *X. laevis* open neural plate stage embryos are shown in transversal sections (Figure 1F and L). *Numb* is found adjacent to the midline in the superficial epidermal layer of the bilayered neuroectoderm, which stays in a proliferative state and primarily undergoes differentiation in the later, secondary wave of neurogenesis [52,53]. In contrast, all three longitudinal expression domains of *NumbL* are located in the deeper sensorial layer of the neuroectoderm, which predominately gives rise to primary neurons. The midline staining of *NumbL* is also located in the deeper sensorial layer that will give rise to the floor plate of the neural tube. In the neural tube of tailbud stage embryos, *Numb* is weakly and broadly expressed (Figure 1H). In contrast, the expression of *NumbL* in the neural tube is stronger and more restricted (Figure 1N). *NumbL* transcripts are excluded from the inner ventricular zone where the proliferating progenitor cells are located and present in the intermediate layer containing differentiating neuronal cells as well as in the outer marginal zone layer.

The temporal expression of *NumbL* in the territories of primary neurogenesis, as well as in the intermediate zone and marginal zone of the neural tube, suggest a role for *NumbL* after cells have initiated differentiation. It was therefore tested if the Neurogenin (Neurog) family of proneural transcription factors can regulate *NumbL* expression in *X. laevis* animal caps. As shown in Figure 1O, *N-tubulin,* a marker of post-mitotic neurons, served as a positive control and was activated by all three Neurogenins. Neurog 1, Neurog2 and Neurog3 also activated *NumbL* but not *Numb,* which is expressed at equal levels in injected and control caps, further supporting a role for *NumbL* during neuronal differentiation downstream of the proneural factors.

The ability of the Neurog2 to regulate *NumbL* expression was also investigated in whole *X. laevis* embryos. *Neurog2* mRNA (20 pg) was coinjected with *β-gal* mRNA (75 pg) into one blastomere of two-cell stage embryos, to localize the distribution of the injected mRNA. As shown by the whole mount in situ hybridization (Figure 1P), Neurog2 ectopically induces the expression of *NumbL* in both the neural and nonneural ectoderm. As shown in the transversal section (Figure 1Q), the ectopic *NumbL expression* is induced only in the deep layer of the ectoderm. As primary neurogenesis is also under negative regulation of the Notch pathway, we investigated if this pathway also influences the expression of *NumbL*. Overexpression of 50 pg mRNA encoding the intracellular domain of Notch (NICD) inhibits *NumbL* expression (Figure 1R and 1S). As the Notch pathway also inhibits *Neurog* expression [30,54], the negative regulation may be an indirect effect. In summary, the expression and regulation of *NumbL* is indicative of a function during neuronal differentiation of the primary neurons.

### NumbL is required for primary neurogenesis in *X. laevis*

**Figure 2:**
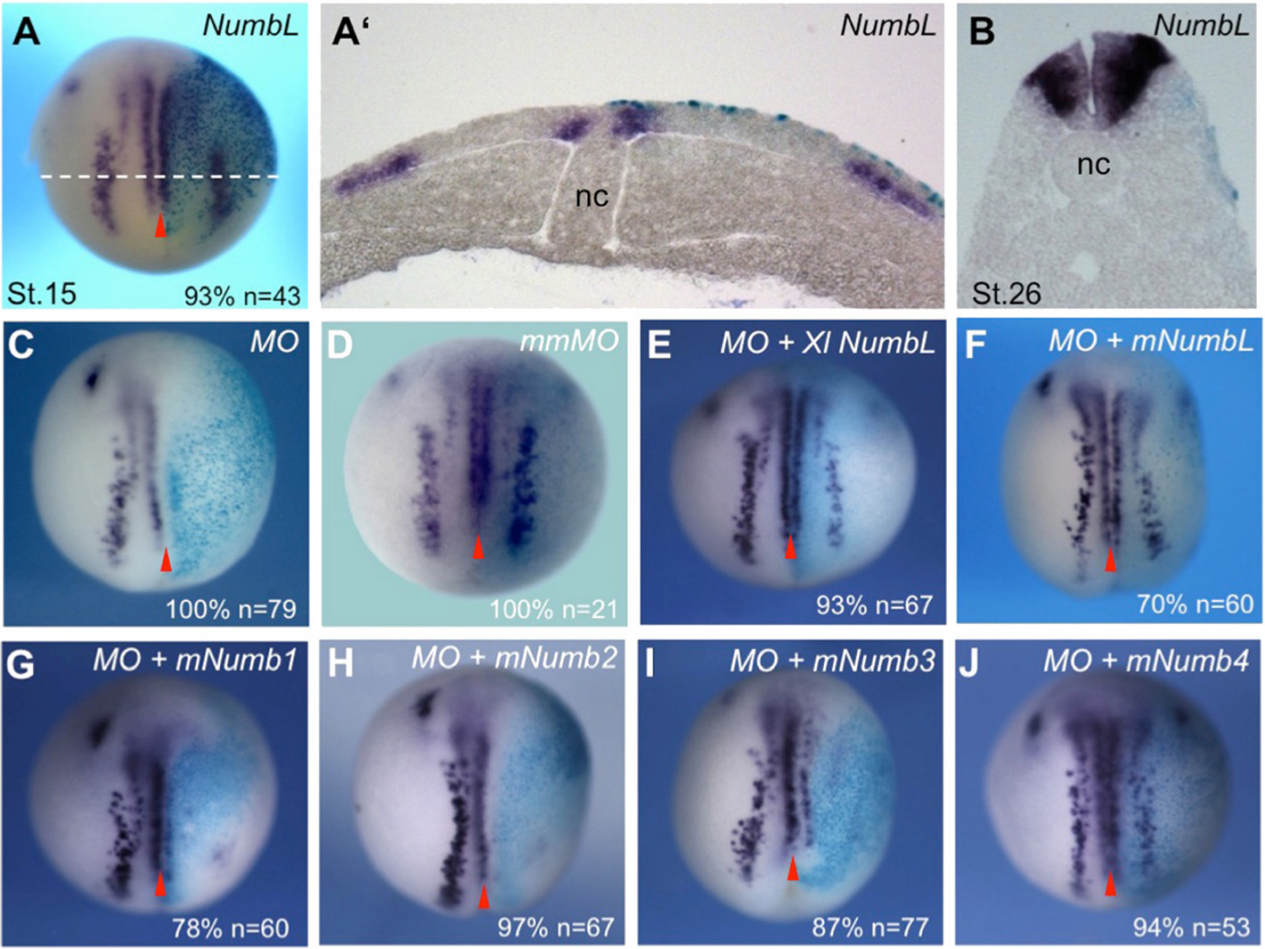
***NumbL *gain and loss of function studies in *X. laevis *embryos. (A**-**B)** Influence of *NumbL* overexpression on neuronal differentiation in *X. laevis* embryos at the open neural plate and tailbud stage. Two cell stage embryos were coinjected animally in one blastomere with *NumbL* mRNA (500 pg) and *β-gal* mRNA (75 pg). The injected embryos were analyzed by whole mount in situ hybridization for *N-tubulin.* Shown is a dorsal view of stage 14 embryos, anterior up **(A)** and the corresponding transversal section **(A')** as well as a transversal section at the level of the hindbrain of a stage 26 embryo **(B)**. **(C**-**J)** Morpholino oligonucleotide mediated knockdown of NumbL. Embryos were injected with 12.5 ng *NumbL MO* or a mismatched MO (mmMO) together with *β-gal* mRNA (75 pg) in one blastomere at the two-cell stage and analyzed by for *N-tubulin* expression at the open neural plate stage. To rescue the *NumbL MO* phenotype, 100 pg of murine *NumbL* or *Numb* mRNA as well as *X. laevis NumbL* mRNA was coinjected as indicated. Embryos are shown in a dorsal view, anterior up and the injected site is marked by X-Gal staining (light blue) and is always on the right. The number of embryos (n) and percentage of embryos showing the described phenotype are indicated in the lower right corner. The midline is indicated with a red arrowhead.

As both the expression and regulation of *NumbL* support a role during primary neurogenesis, an analysis of *NumbL* function in this context was performed. In a first approach, mRNA encoding a Myc-tagged version of *NumbL (MT-NumbL)* was overexpressed in *X. laevis* embryos and the influence on primary neuron formation was monitored by whole mount in situ hybridization (Figure 2A-B). At the open neural plate stage, no significant increase in density of *N-tubulin* positive cells was observed (Figure 2A). However, in transverse sections of tailbud stage embryos overexpressing *NumbL*, an increase in the size of the neural tube with a concomitant increase in *N-tubulin* expression is observed (Figure 2B). It is unclear if the increase in *N-tubulin* staining is a direct influence of NumbL on differentiation of the neuronal progenitor cells or the result of increased proliferation.

To further study the role of *NumbL* in the context of primary neurogenesis, an antisense morpholino oligonucleotide (MO) was designed to inhibit translation of the endogenous *NumbL* mRNA. Functionality and specificity was demonstrated by the ability of the *NumbL-MO*, but not a 5 bp mismatch MO (mmMO), to inhibit the translation of NumbL in an *in vitro* translation assay and of a GFP reporter harbouring the 5′-UTR of *NumbL* in *X. laevis* embryos (Additional file 6: Figure S6).

Injection of the *NumbL-MO* (12.5 ng), but not of the *NumbL mmMO*, strongly inhibited *N-tubulin* expression on the injected side (Figure 2C and D). The loss of neuronal differentiation by the *NumbL-MO* could be rescued by co-injection of *X. laevis NumbL* mRNA, which lacks the endogenous 5′-UTR and is therefore not targeted by MO (Figure 2E). The *NumbL-MO* phenotype could also be rescued by coinjection with the mouse *NumbL* mRNA (Figure 2F). The ability of the mouse Numb isoforms to rescue the NumbL MO phenotype was also investigated (Figure 2G-J). Interestingly*, mNumb4* was the only *mNumb* isoform able to rescue the loss of NumbL in *X. laevis* embryos. Numb4, similar to NumbL, lacks the inserts in both the PTB and the PRR domains.

### NumbL knockdown does not cause a de-regulation of the Notch signaling pathway

**Figure 3:**
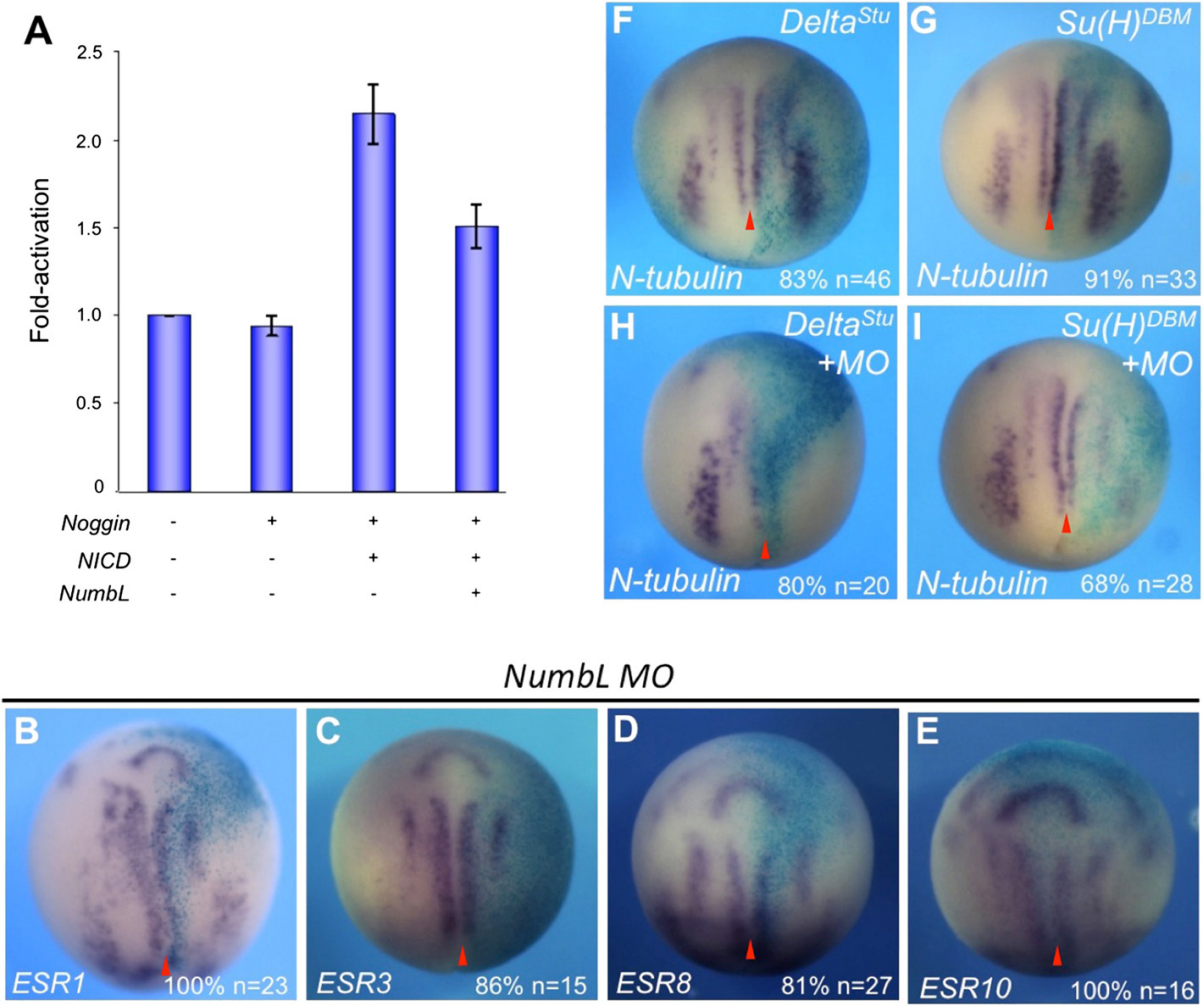
***X. laevis *NumbL inhibits Notch signaling in reporter assays, but knockdown of NumbL does not influence the expression of Notch target genes in the open neural plate. (A)** The influence of *NumbL* overexpression on a Notch responsive reporter. *X. laevis* embryos were injected with the *Notch-ICD (NICD)*, *Noggin* and *NumbL* mRNA as indicated, together with a *Hes1*-luciferase reporter. Embryos were cultivated until open neural plate stage and luciferase activity was measured and normalzed to renilla luciferase. Shown is a summary of four independent luciferase experiments. Two batches of embryos containing at least ten embryos per injection mix were collected for each experiment. The error bars represent the standard deviation. **(B**-**E)***NumbL* MO (12.5 ng) does not lead to an increase in Notch target gene expression at open neural plate stages. **(F**-**I)** The *NumbL* MO induced loss of neuronal differentiation is not rescued by inhibition of Notch signaling. *Delta*^*Stu*^ or *Su(H)*^*DBM*^ mRNA was injected alone or in combination with 12.5 ng NumbL MO into one blastomere at the two-cell stage and *N-tubulin* expression analyzed by whole mount in situ hybridization at stage 14. Whole mount in situ probes are indicated in the lower left corner. Embryos are shown in a dorsal view, anterior up. The injected side is marked by X-Gal staining and is always on the right. The midline is indicated with a red arrowhead.

One of the best-described functions for members of the Numb family is their ability to act as an inhibitor of Notch signaling [4,5]. The loss of neuronal differentiation observed upon knock-down of NumbL in *X. laevis* may therefore be the result of increased Notch signaling, which would then suppress neurogenesis. We first addressed the ability of NumbL to inhibit Notch signaling using a Notch responsive Hes1-luciferase reporter in *X. laevis* embryos. The reporter was injected into both blastomeres of the two-cell stage embryos together with the indicated mRNAs, and at stage 15 the embryos luciferase activity was measured using the dual luciferase assay. Injection of NICD mRNA gave only a mild activation of the Hes1-luciferase reporter (data not shown). To obtain higher reporter activity, mRNA encoding the neural inducer *Noggin* that acts through inhibiting BMP signaling, was co-injected with *NICD* mRNA. As shown in Figure 3A, coinjection of *NICD* and *Noggin* mRNA induced the reporter more than 2-fold; indeed, coinjection of *NumbL* mRNA reduced Notch-mediated induction of the reporter.

To test, whether a knockdown of NumbL would lead to increased Notch activity during primary neurogenesis, Notch target genes that are expressed in the open neural plate were evaluated in *NumbL MO* injected embryos (Figure 3B-E). Surprisingly, in *NumbL MO* injected embryos, no increase in *ESR1, 3, 8 or 10* expression was observed. The loss of *N-tubulin* expression by the *NumbL MO* could also not be rescued by blocking the Notch pathway through co-expression of dominant negative mutants of *Suppressor of Hairless* (*Su(H)*^*DBM*^) or *Delta* (*Delta*^*STU*^) (Figure 3F-I). These data suggest that, even though NumbL has the intrinsic ability to act as a Notch inhibitor, the inhibition of primary neurons upon knockdown of NumbL in *X. laevis* embryos may be the result of a Notch pathway independent mechanism.

### NumbL knockdown increases neural gene expression and inhibits neuronal differentiation

**Figure 4:**
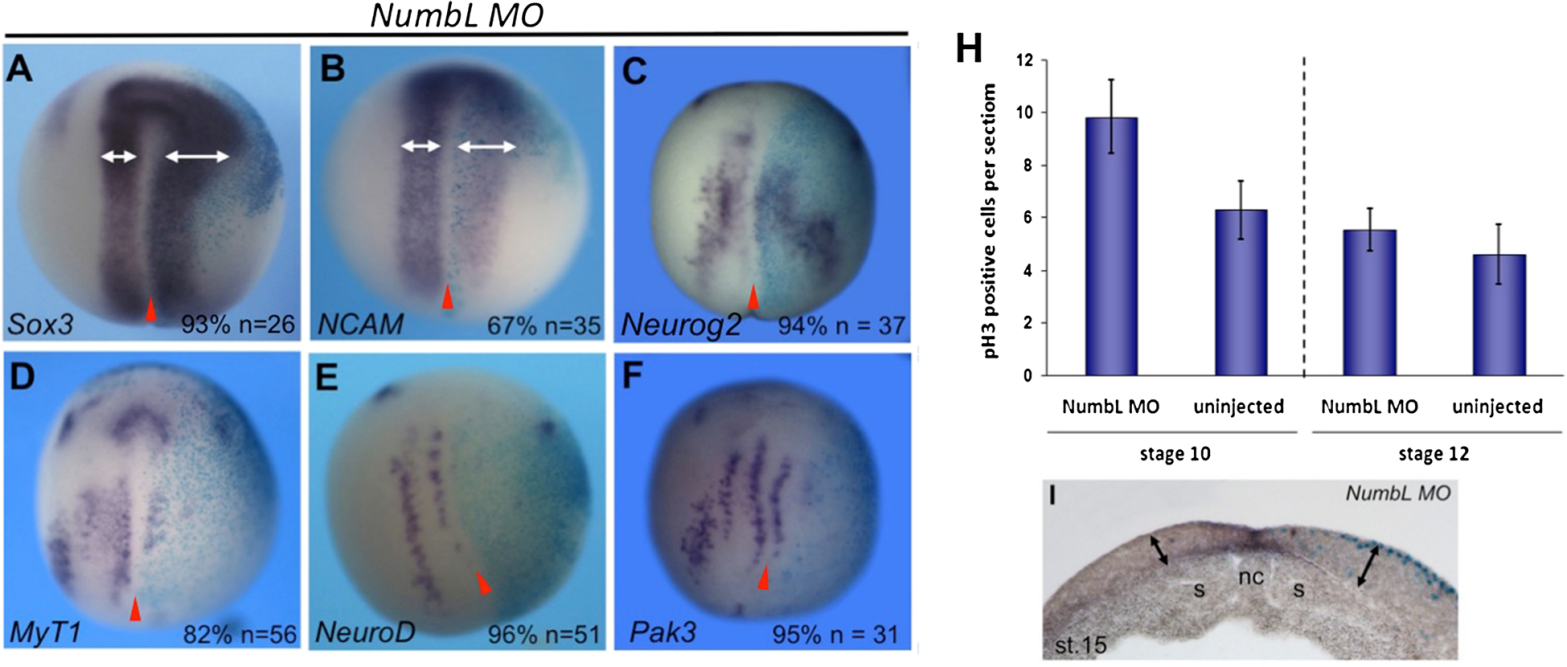
**Influence of NumbL knockdown on pan-neural and neuronal differentiation genes as well as proliferation. (A**-**F)***X. laevis* embryos were injected into one blastomere at the two-cell stage with of 12.5 ng of NumbL MO together with *β-gal* mRNA (75 pg), and analyzed at the open neural plate stage for the indicated markers by whole mount in situ hybridization. Embryos are shown in a dorsal view, anterior up; the injected side marked by X-Gal staining is always on the right. Statistics are indicated in the lower right corner. **(H)** Control embryos and embryos injected with 12.5 ng of *NumbL MO*, were collected at stage 10 and stage 12 and mitotically active cells visualized by staining for pH3. The graph shows the statistical evaluation of 15 consecutive sections of two embryos per stage, based on the number of pH3 positive cells in a defined area on the injected and uninjected site as indicated in the cross-sections. **(I)** Black double arrows indicated the size of the neural epithelium on the control side (left) and on the injected side (right) from stage 15 embryos. The midline is indicated with a red arrowhead. s, somite, nc, notochord.

To determine, whether the observed loss of postmitotic neurons upon NumbL knockdown in *X. laevis* embryos was the consequence of alterations affecting the neural progenitor pool, expression of the early neural genes *Sox3* and *NCAM* was evaluated in NumbL knockdown embryos (Figure 4A and B). The expression domains of *Sox3* and *NCAM* were found to be expanded on the side of the embryo injected with the *NumbL MO*. Thus, knockdown of NumbL may keep the neural progenitor cells in a proliferative state and therefore impair neuronal differentiation. To elucidate, whether the expansion of progenitors markers would result from increased proliferation in the neuroectoderm, we compared the number of mitotically active cells in the neuroepithelium by phosphorylated Histone 3 (pH3) staining. While, at stage 12, only a slight increase in pH3 positive cells could be detected, a significant increase in mitotically active cells in the prospective neuroectoderm was observed at the onset of gastrulation at stage 10 (Figure 4H), which may result in the thickened neural epithelium (Figure 4I).

Differentiation of neural progenitor cells requires withdrawal from the cell cycle, which is achieved by the expression of cell cycle regulators such as *Pak3* and p27^Xic1^[44,55]. Consistent with the increase in mitotically active cells, a loss of *Pak3* expression was observed in stage 15 NumbL morphants (Figure 4F), thus raising the possibility that the neuronal progenitors are prevented from undergoing differentiation because they are not able to exit the cell cycle.

As an expansion of neural progenitors and a loss of cell cycle regulators were detected upon knockdown of NumbL, the question arose whether neuronal differentiation was inhibited due to a loss of essential neuronal determination factors. We therefore evaluated the influence of NumbL knockdown on the neuronal determination gene *Neurog2* in *X. laevis* embryos by whole mount in situ hybridization. Upon injection of *NumbL MO*, *Neurog2* expression was still present, however, the expression domains appeared slightly dispersed (Figure 4C). However, this cannot explain the complete loss of *N-tubulin* expression. In contrast to the weak influence on *Neurog2*, downstream differentiation markers, such as *NeuroD*[45] and *MyT1*[42] were found to be more strongly inhibited (Figure 4D and E), suggesting that the neuronally determined cells expressing *Neurog2* are prevented from undergoing differentiation on this downstream level.

### NumbL interacts with the AP-2 complex in open neural plate stage embryos

**Figure 5:**
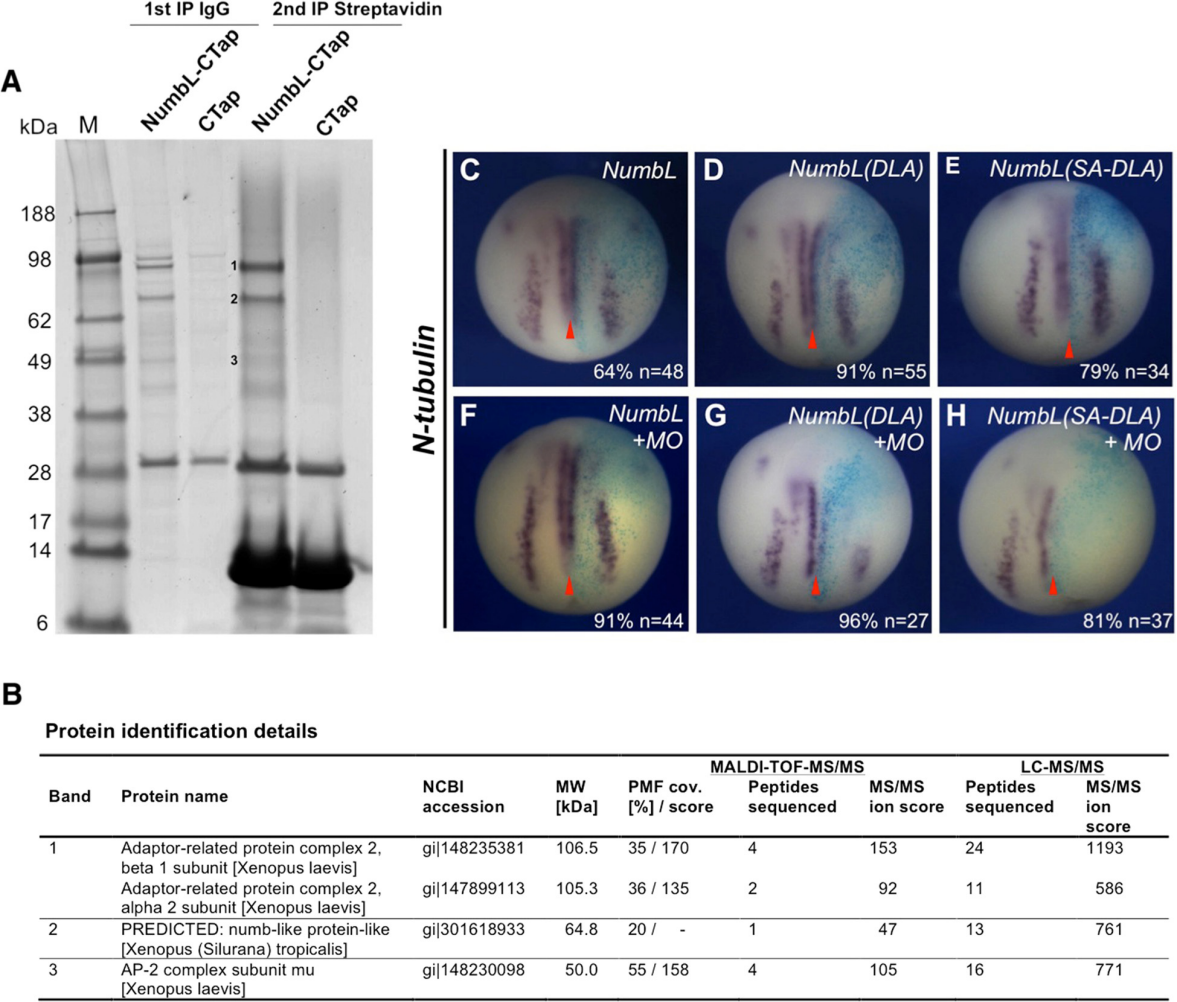
**NumbL interacts in a tandem affinity purification approach with subunits of the AP-2 complex. (A)** Colloidal coomassie blue stained gradient gel of two subsequent immunoprecipitations from *X. laevis* embryos injected in both blastomeres with 500 pg of *NumbL-CTap* or *CTap* mRNA. The bands representing NumbL and major specifically co-immunoprecipitated proteins (i.e. *Xenopus* proteins that were identified in the NumbL-CTap, but not in the CTap lane) are indicated on the gel as follows: 1, AP-2 beta 1 subunit, AP-2 alpha 2 subunit; 2, NumbL; 3, AP-2 mu1 subunit. **(B)** Table listing the protein identification details, the numbering of the bands refers to the gel image in Figure 5A. The number of peptides sequenced refers to the set of non-redundant peptides that have been assigned to the protein sequence according to the MASCOT MS/MS ion search algorithm. **(C**-**H)** Putative AP-2 binding mutants of NumbL are not able to rescue the MO phenotype. Embryos were injected into one blastomere at the two-cell stage with 500 pg of *NumbL* mRNA and 12.5 ng *NumbL MO* as indicated in the upper right corner together with *β-gal* mRNA (75 pg) and *N-tubulin* expression was analyzed by whole mount in situ hybridization. Shown are stage 15 embryos in a dorsal view, anterior up. The injected site is marked by X-Gal staining (light blue) and is always on the right. The number of embryos (n) and percentage of embryos showing the described phenotype are indicated in the lower right corner. The midline is indicated with a red arrowhead.

Numb family proteins contain multiple protein interaction domains and are thought to act as scaffold proteins [3]. In order to identify NumbL interaction partners during early neural plate stages, a tandem affinity purification approach was undertaken using a dual-tagged version of NumbL (NumbL-CTap). *NumbL-CTap* or *CTap* mRNA, which served as a negative control, were injected into both blastomeres at the two-cell stage, embryos cultivated until stage 15. In two subsequent pull-down experiments from cell lysates, NumbL-CTap and CTap interacting proteins were isolated and specific Numb-CTap binding proteins identified by mass spectrometry (Figure 5A and B). Three proteins were identified by MALDI fingerprinting and LC-MS/MS sequencing. All three proteins were subunits of the clathrin-associated adaptor-related protein 2 (AP-2) complex, α2, β1 and μ1. These results reveal the ability of NumbL to interact with the components of the endocytotic machinery when primary neurons are differentiating in *Xenopus* embryos.

To determine if the identified interaction of NumbL with the AP-2 complex was of functional relevance, mutants of NumbL were designed. In Numb, mutation of the DPF motif to DLA has been shown to abolish Numb binding to AP-2 [3]. Therefore, the corresponding motif in NumbL, the DQF motif, was mutated to DLA. In addition, since phosphorylation of Numb was shown to impair binding to the AP-2 complex, we created a NumbL phosphorylation mutant, NumbL(SA-DLA), as well. Overexpression of *NumbL(DLA*) and *NumbL(SA-DLA)* mRNA did not significantly increase *N-tubulin* staining on the injected side (Figure 5C-E). To analyze for the necessity of the AP-2 interaction in NumbL function, rescue experiments for the NumbL MO effects were done. In these experiments, in contrast to the wild-type *NumbL,* both *NumbL* mutants could not rescue the MO phenotype (Figure 5F-H). These results suggest that the interaction with the AP-2 complex is important for the activity of NumbL during *X. laevis* neuronal differentiation.

## Discussion

The establishment of the vertebrate nervous system is a tightly regulated process controlled through multiple intrinsic and extrinsic regulators. The adaptor proteins Numb and NumbL have been implicated in the developmental of the nervous system, however the molecular mechanism of their action remains controversial. The loss of both *Numb/NumbL* in mice has strong influences on the neural progenitor cell population [22,24,25]. Dependent on the stage of development when gene inactivation occurs, either depletion of neural progenitors as a consequence of premature differentiation, hyperproliferation of the neural progenitors or impaired differentiation of the progenitor population was observed. We therefore analyzed the expression of *Numb* and *NumbL* during *Xenopus* embryogenesis, where the first primary neurons are born in the open neural plate in a stereo- and temporal-specific manner.

As shown by whole mount in situ hybridization, *NumbL* transcripts are present in open neural plate stage *X. laevis* and *X. tropicalis* embryos in the three bilateral stripes, where primary neurons are known to arise. The temporal expression of *NumbL* in these territories and exclusion from the inner ventricular zone of the spinal cord argue for a role of *NumbL* after the onset of differentiation. In contrast, the spatial expression of *Numb* transcripts is not indicative of a role during primary neurogenesis. In fact, in open neural plate stage embryos, *Numb* was present in the superficial layer of the neuroepethilium, which is refractory to primary neurogenesis [52]. In the developing nervous system of mice, chicken and zebrafish, *Numb* is broadly expressed and is also found in the mitotically active progenitor cells within the neural epithelium, while the expression of *NumbL* is restricted to the developing nervous system and found in post-mitotic differentiating cells [10,11,15,18]. The similarity observed in respect to the expression patterns of *Numb* and *NumbL* within different vertebrate species strongly argues for a conservation of function in the context of neurogenesis. In addition to the expression of *NumbL* in the differentiating neurons, we also observed an earlier expression in the dorsal ectoderm of gastrula stage embryos. This suggests that during the development of the *Xenopus* nervous system, *NumbL* may serve different roles, one during the establishment of the neural ectoderm and a later function in the process of neuronal differentiation.

Both, the spatial expression of *Xenopus NumbL* and its positive regulation by the proneural transcription factor Neurog2, suggest a role in the context of primary neurogenesis. This was further supported by the complete loss of postmitotic neurons at the open neural plate stage upon knockdown of *NumbL* and demonstrates that *NumbL* is essential for primary neurogenesis. While single knockouts of *NumbL* in the mouse did not reveal a significant phenotype, conditional knockout of *Numb* in a *NumbL* null background results in embryonic lethality and a loss of differentiated neurons at E10.5, which is a much stronger phenotype compared to loss of *Numb* alone [23-25]. A failure to observe a neural phenotype in mouse *NumbL* knockouts might be due to the incompleteness of the knockdown, as low levels of *NumbL* are still detectable in the homozygous mutants [23].

The NumbL morpholino-induced loss of neuronal differentiation could be rescued by mouse NumbL and Numb4, but not by any of the other Numb isoforms. Interestingly, Numb4 is structurally most similar to NumbL, lacking inserts within both the PTB and PRR domain. This further lends support to the notion that the different structural isoforms of Numb and NumbL have distinct activities, giving rise to the diversity of functions attributed to the Numb proteins [12]. Numb PRR_L_ isoforms promote proliferation of a variety of progenitor cells including neuroepithelial stem cells of the *Drosophila* larval brain, P19 embryonic carcinoma cells as well as primary and immortalized neural crest stem cell lines [19,50,56]. In contrast, PRR_S_ isoforms inhibit proliferation and promote neuronal differentiation of these same cells [19,50,56]. The presence or absence of the PTB insert did not significantly contribute to the ability of the Numb isoforms to influence the proliferation and differentiation of the progenitor cells [50]. However, the length of the PTB domain, but not of the PRR domain, was found to influence neurite outgrowth in response to neurotrophic factors and cell survival upon withdrawal [23]. The PTB domain was also found to be responsible for controlling the subcellular localization of Numb. In mammalian cell lines, Numb isoforms that do not have the 11 amino acid insert in the PTB (PTB_S_) were localized to the plasma membrane, while the PTB_L_ Numb isoforms and NumbL were found to localize symmetrically in the cytoplasm [9,11,13,50]. We also observed that NumbL localizes to the cytoplasm of *X. laevis* ectodermal explants and HeLa cells (data not shown).

Microinjection of *NumbL* mRNA into *X. laevis* early cleavage stage embryos is not sufficient to promote ectopic formation of primary neurons, but leads to a slight increase in neuronal density in the territories of primary neurogenesis at the open neural plate stage. Numb and NumbL are modified at the post-translational level, which may contribute to the inability to obtain a strong gain-of-function phenotype. Serine phosphorylation of Numb and NumbL facilitates their binding to the 14-3-3 protein and disrupts their interaction with the clathrin-associated adapter AP-2 [57,58]. However, overexpression of a phosphorylation mutant of NumbL, in which the equivalent serine phosphorylation residues were mutated to alanine, did not result in a significantly stronger phenotype than wild-type gain-of-function. The translation of *NumbL* is also inhibited by miR-34a and miR-184 in murine neural progenitor cells [59,60]. Inhibition of the injected NumbL mRNA by these miRNAs is unlikely, as their target sites are located in the NumbL 3′-UTR, which was not present in the injected mRNA. Alternatively, since NumbL functions as a scaffold protein, its activity is most likely dependent on the availability of its interaction partners. Therefore, the weak phenotype observed upon NumbL overexpression may also be explained by limiting amounts of interacting proteins.

One of the best-characterized functions of the Numb family of proteins is its ability to inhibit Notch signaling [6,8]; correspondingly, in luciferase reporter assays, NumbL attenuated NICD-mediated activation of a luciferase reporter in *X. laevis* embryos. As *X. laevis* primary neuron formation is under negative control of the Notch signaling pathway [32], it was anticipated that the loss of primary neurons observed upon morpholino knock-down of NumbL was due to an increase in Notch signaling. Surprisingly, Notch direct target genes were not activated upon a loss of NumbL. Moreover, inhibition of Notch signaling through the use of dominant negative versions of the ligand Delta and the transcription factor Su(H) could not rescue the NumbL morpholino phenotype. Taken together, we obtained no evidence that NumbL acts as an inhibitor of Notch signaling in the context of primary neurogenesis and suggest a function of NumbL during this process that is Notch-independent.

Mouse and human Numb and NumbL interact directly with components of the endocytotic machinery, namely α-adaptin, a component of the AP-2 complex, and Eps15 via C-terminal DPF and NPF motifs, respectively [3]. The Numbs have been shown to participate in endocytosis and post-endocytic trafficking of several proteins including Notch, integrin, E-cadherin and APP [2,20,61,62]. Although the previously characterized α-adaptin interaction motif in *X. laevis and X. tropicalis* Numb is not conserved and is exchanged to a DQF motif (Additional file 6: Figure S6), components of the AP-2 complex were found to interact with NumbL in open neural plate stage in *X. laevis* embryos. A *NumbL* construct in which the putative alpha-adaptin interaction domain was mutated (Numb-DLA) failed to rescue the loss of neuronal differentiation by knockdown of NumbL, which suggests that this activity may be required for primary neuron formation.

## Conclusion

Loss of function studies demonstrate *X. laevis* NumbL is required for the differentiation of primary neurons born at the open neural plate stage. As loss of NumbL did not influence Notch signaling at this stage, it will be of interest for future studies to identify the pathways and factors regulated by NumbL. This is important not only due to the requirement of *NumbL* during *X. laevis* neurogenesis, but also the emerging role of *NumbL* in tumorgenesis [63].

## Competing interests

The authors declare that they have no competing interests.

## Authors’ contributions

FN conducted most of the experimental work together with MH. OJ performed the mass spectrometry analysis. KH performed some experimental work and wrote the final manuscript. KH and TP designed and coordinated the experimental work. All authors contributed to the manuscript preparation and have read and approved its final version.

## Supplementary Material

Additional file 1: Figure S1 Alignment of the NumbL predicted amino acid sequences from *X. laevis (Xl)* (KF589315), *X. tropicalis (Xt)* (XP_002938862) and *Mus musculus (m)* (NP_035080). Alignment was done using Cluster V method using the DNA Star Lasergene Megalign program. Identical conserved amino acids are highlighted in yellow, the blue bar indicates the putative PTB domain, the green bar marks a Q15 repeat and the orange bars mark the α-Adaptin binding motif DPF (DQF) and the Eps-15 binding motif NPF, respectively. Click here for file

Additional file 2: Figure S2 Alignment of the mouse Numb 1–4 protein sequences with the predicted amino acid sequences from *X. tropicalis (Xt) Numb* (NM_001097359). The following mouse reference sequences were used: Numb 1/p66 (NP_001129547.1), Numb2/p72 (NP_035079.1) Numb3/p71 (NP_001258984.1) and Numb4/p65 (NP_001258985.1). Alignment was done using Cluster V method using the DNA Star Lasergene Megalign program. Identical conserved amino acids are highlighted in yellow, the blue bar indicates the putative PTB domain, the green bar marks the insert in the PRR domain and the orange bars mark the alpha-Adaptin binding motif DPF (DQF) and the Eps-15 binding motif NPF, respectively. Click here for file

Additional file 3: Figure S3 Comparative whole mount in situ expression analysis of staged *X. laevis* embryos using an antisense (A-D) and sense (E-H) *X. tropicalis Numb* RNA *probe*. Stage 11 embryos shown in A and E are a blastopore view. Stage 16 embryos are shown in an anterior view (B and F) and a dorsal view anterior down (C and G). Stage 26 embryos are shown in a lateral view (D and H) anterior right. Click here for file

Additional file 4: Figure S4 Double whole mount in situ hybridization expression analysis of stage 15 *X. laevis* embryo with an antisense *X. tropicalis Numb* probe (dark purple) and *X. laevis* EpiK (red). (A) Shown is a dorsal view, anterior down. The blue arrowheads mark the beginning of the longitudinal stripe of *Numb* expression. (B) Transversal section of the embryo depicted in (A). *Numb* expression (blue bracked) in the superficial layer of the ectoderm is directly flanked laterally by *EpiK* expression (onset indicated by red arrow), which is excluded from the neural ectoderm. Click here for file

Additional file 5: Figure S5 Comparative whole mount in situ expression analysis of staged *X. tropicalis* embryos using an antisense *X. tropicalis Numb3* RNA probe (A-I) or a antisense *X. laevis NumbL* RNA probe (J-R). The embryos in A, H, I, J, Q, and R are shown in a lateral view; in B, D, F, K, M and O in an anterior view; in C, E, G, L, N and P in a dorsal view. The expressions patterns obtained are highly correlative with those shown Figure 1 using *X. laevis* embryos demonstrating cross-species probe hybridization of *Numb3* and *NumbL* and their conservation in expression. Click here for file

Additional file 6: Figure S6 Verification of the NumbL MO function *in vitro* and *in vivo.* (A) The *NumbL MO* (MO) but not the NumbL mismatch MO (mmMO) inhibits translation of *NumbL* in in vitro assays. Per reaction, 500 μg NumbL-pCS2 and 1000 ng, 100 ng or 10 ng of MO were used and analyzed by 12% SDS-PAGE. (B): The *NumbL MO* inhibits GFP reporter construct activities in *X. laevis* embryos. Embryos were injected in both blastomeres of the two-cell stage with 100 pg of mRNA encoding for the NumbL-5′UTR-GFP reporter and 5 ng of MO. GFP expression was evaluated at stage 10.5. A schematic representation of the reporter construct is shown below, MO binding site is indicated. Click here for file
