# Supplementary figures and images for: NumbL is essential for Xenopus primary neurogenesis
Source: BMC Dev Biol. 2013 Oct 14;13:36. doi: 10.1186/1471-213X-13-36 (PMC3852787; doi:10.1186/1471-213X-13-36)

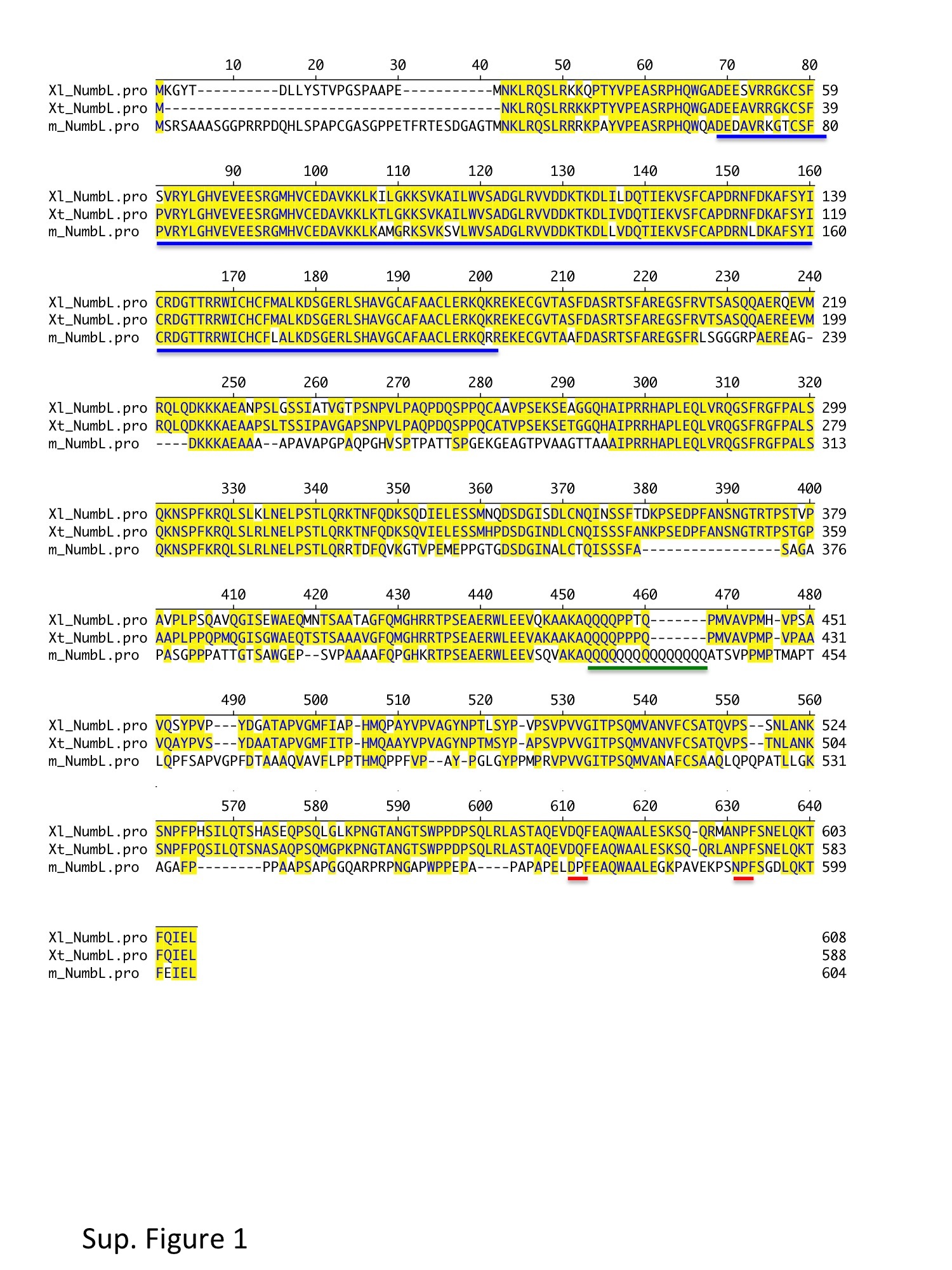

Supplement: Additional file 1: Figure S1 — Alignment of the NumbL predicted amino acid sequences from X. laevis (Xl) (KF589315), X. tropicalis (Xt) (XP_002938862) and Mus musculus (m) (NP_035080). Alignment was done using Cluster V method using the DNA Star Lasergene Megalign program. Identical conserved amino acids are highlighted in yellow, the blue bar indicates the putative PTB domain, the green bar marks a Q15 repeat and the orange bars mark the α-Adaptin binding motif DPF (DQF) and the Eps-15 binding motif NPF, respectively. [file 1471-213X-13-36-S1.jpeg]

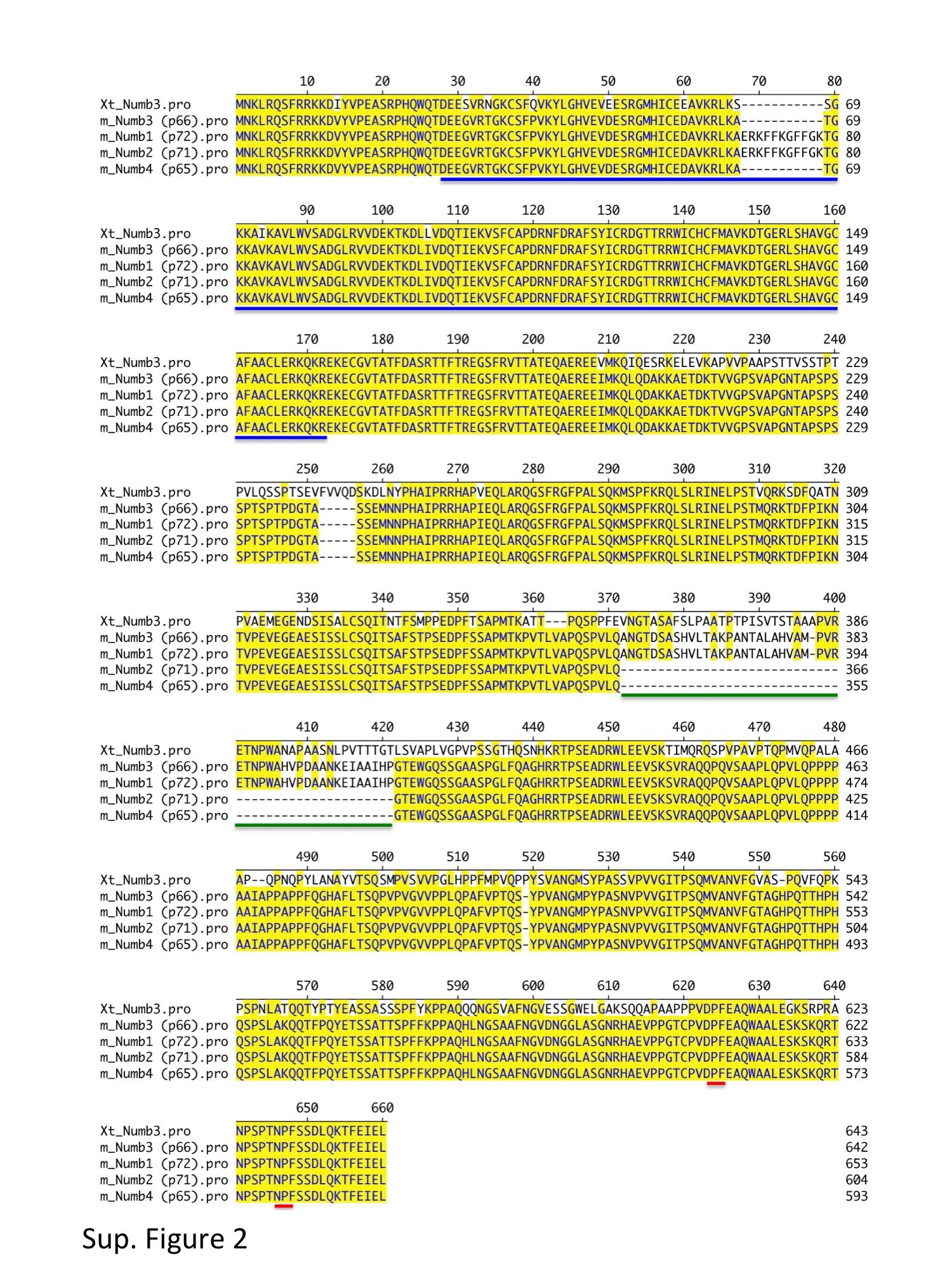

Supplement: Additional file 2: Figure S2 — Alignment of the mouse Numb 1–4 protein sequences with the predicted amino acid sequences from X. tropicalis (Xt) Numb (NM_001097359). The following mouse reference sequences were used: Numb 1/p66 (NP_001129547.1), Numb2/p72 (NP_035079.1) Numb3/p71 (NP_001258984.1) and Numb4/p65 (NP_001258985.1). Alignment was done using Cluster V method using the DNA Star Lasergene Megalign program. Identical conserved amino acids are highlighted in yellow, the blue bar indicates the putative PTB domain, the green bar marks the insert in the PRR domain and the orange bars mark the alpha-Adaptin binding motif DPF (DQF) and the Eps-15 binding motif NPF, respectively. [file 1471-213X-13-36-S2.jpeg]

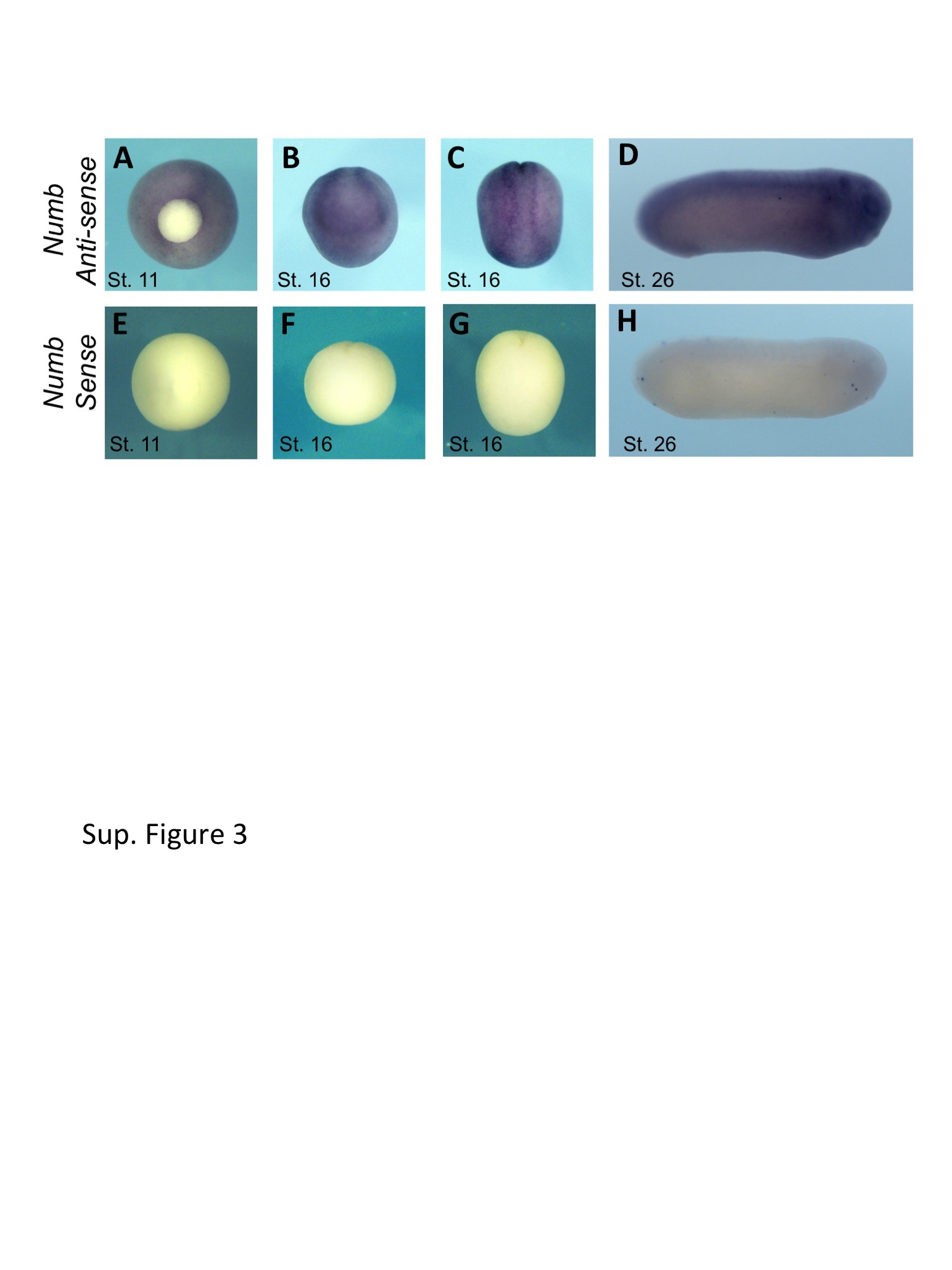

Supplement: Additional file 3: Figure S3 — Comparative whole mount in situ expression analysis of staged X. laevis embryos using an antisense (A-D) and sense (E-H) X. tropicalis Numb RNA probe. Stage 11 embryos shown in A and E are a blastopore view. Stage 16 embryos are shown in an anterior view (B and F) and a dorsal view anterior down (C and G). Stage 26 embryos are shown in a lateral view (D and H) anterior right. [file 1471-213X-13-36-S3.jpeg]

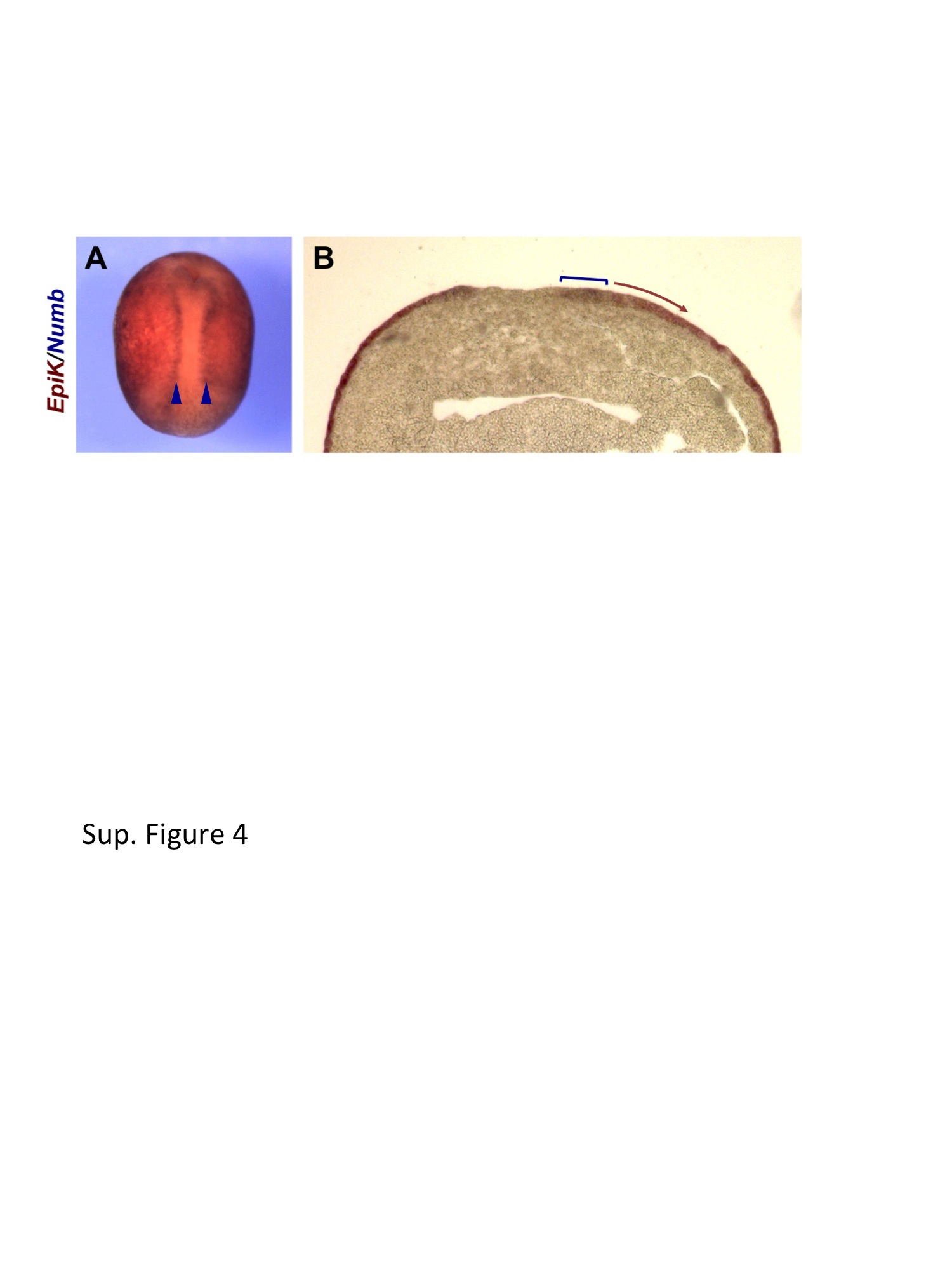

Supplement: Additional file 4: Figure S4 — Double whole mount in situ hybridization expression analysis of stage 15 X. laevis embryo with an antisense X. tropicalis Numb probe (dark purple) and X. laevis EpiK (red). (A) Shown is a dorsal view, anterior down. The blue arrowheads mark the beginning of the longitudinal stripe of Numb expression. (B) Transversal section of the embryo depicted in (A). Numb expression (blue bracked) in the superficial layer of the ectoderm is directly flanked laterally by EpiK expression (onset indicated by red arrow), which is excluded from the neural ectoderm. [file 1471-213X-13-36-S4.jpeg]

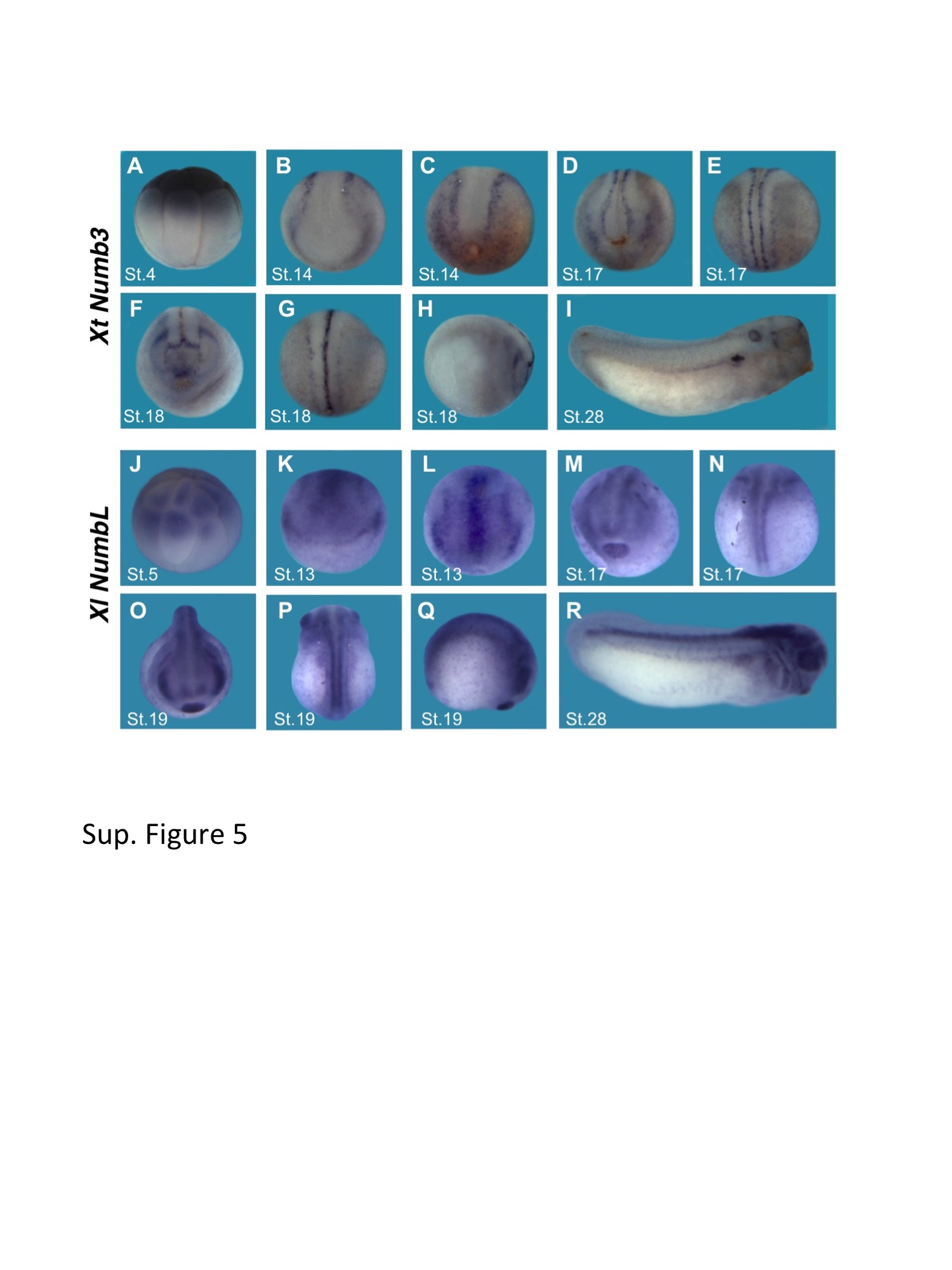

Supplement: Additional file 5: Figure S5 — Comparative whole mount in situ expression analysis of staged X. tropicalis embryos using an antisense X. tropicalis Numb3 RNA probe (A-I) or a antisense X. laevis NumbL RNA probe (J-R). The embryos in A, H, I, J, Q, and R are shown in a lateral view; in B, D, F, K, M and O in an anterior view; in C, E, G, L, N and P in a dorsal view. The expressions patterns obtained are highly correlative with those shown Figure 1 using X. laevis embryos demonstrating cross-species probe hybridization of Numb3 and NumbL and their conservation in expression. [file 1471-213X-13-36-S5.jpeg]

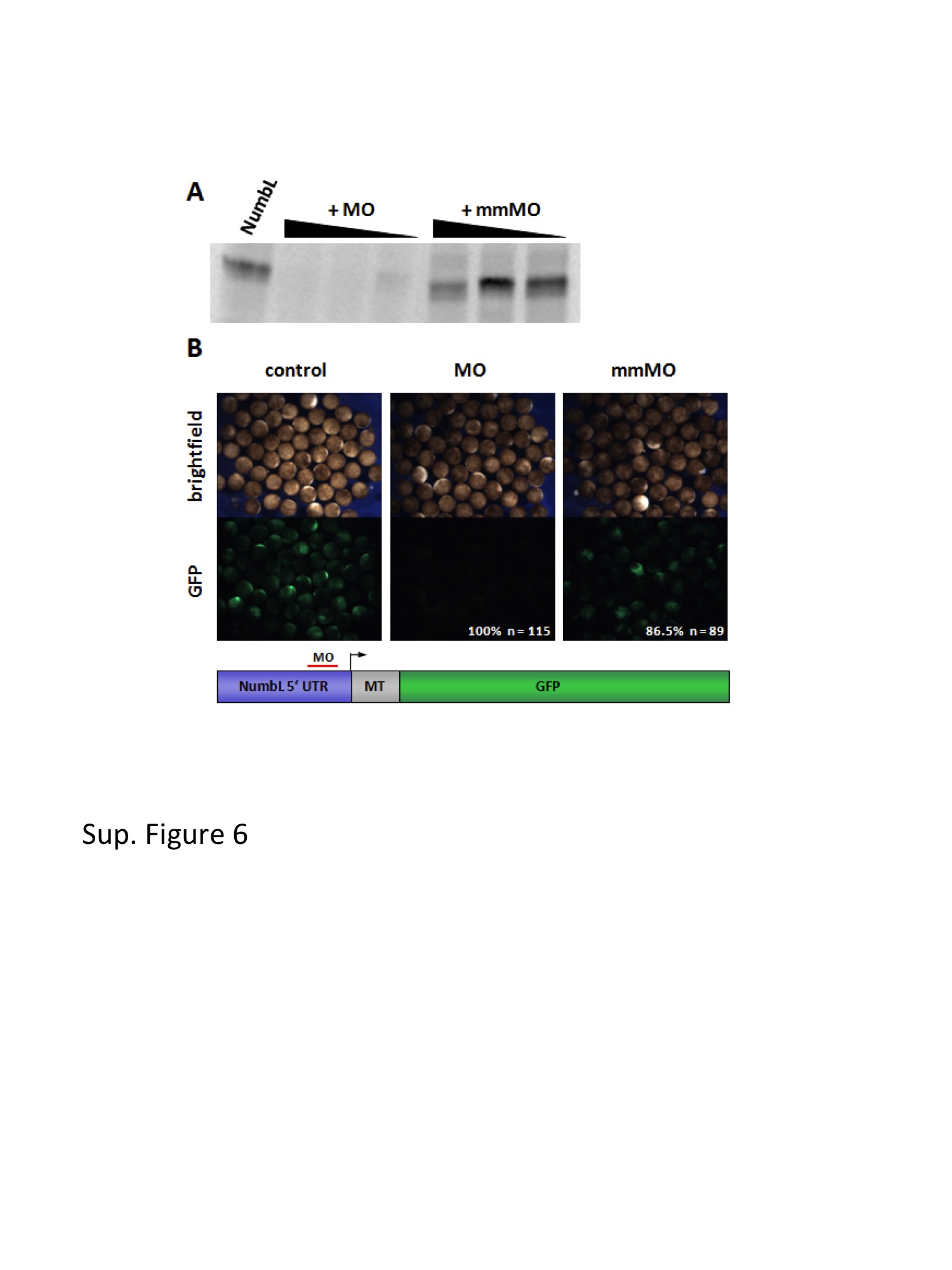

Supplement: Additional file 6: Figure S6 — Verification of the NumbL MO function in vitro and in vivo. (A) The NumbL MO (MO) but not the NumbL mismatch MO (mmMO) inhibits translation of NumbL in in vitro assays. Per reaction, 500 μg NumbL-pCS2 and 1000 ng, 100 ng or 10 ng of MO were used and analyzed by 12% SDS-PAGE. (B): The NumbL MO inhibits GFP reporter construct activities in X. laevis embryos. Embryos were injected in both blastomeres of the two-cell stage with 100 pg of mRNA encoding for the NumbL-5′UTR-GFP reporter and 5 ng of MO. GFP expression was evaluated at stage 10.5. A schematic representation of the reporter construct is shown below, MO binding site is indicated. [file 1471-213X-13-36-S6.jpeg]
